# C,C- and C,N-Chelated Organocopper Compounds

**DOI:** 10.3390/molecules26195806

**Published:** 2021-09-25

**Authors:** Liang Liu, Hui Chen, Zhenqiang Yang, Junnian Wei, Zhenfeng Xi

**Affiliations:** 1 Beijing National Laboratory for Molecular Sciences (BNLMS), Key Laboratory of Bioorganic Chemistry and Molecular Engineering of Ministry of Education, College of Chemistry, Peking University, Beijing 100871, China; liang.liu@pku.edu.cn; 2 Henan Institute of Chemistry Co., Ltd., Henan Academy of Sciences, Zhengzhou 450002, China; hchen@kingorgchem.com (H.C.); yangzq@chuanganchem.com (Z.Y.); 3 State Key Laboratory of Organometallic Chemistry, Shanghai Institute of Organic Chemistry (SIOC), Shanghai 200032, China

**Keywords:** organocopper, C,N-chelated, C,C-chelated, bidentate, butadienyl, intramolecular coordination, three center-two electron bonds, structure-reactivity relationship

## Abstract

Copper-catalyzed and organocopper-involved reactions are of great significance in organic synthesis. To have a deep understanding of the reaction mechanisms, the structural characterizations of organocopper intermediates become indispensable. Meanwhile, the structure-function relationship of organocopper compounds could advance the rational design and development of new Cu-based reactions and organocopper reagents. Compared to the mono-carbonic ligand, the C,N- and C,C-bidentate ligands better stabilize unstable organocopper compounds. Bidentate ligands can chelate to the same copper atom via *η*^2^-mode, forming a mono-cupra-cyclic compounds with at least one acute C-Cu-C angle. When the bidentate ligands bind to two copper atoms via *η*^1^-mode at each coordinating site, the bimetallic macrocyclic compounds will form nearly linear C-Cu-C angles. The anionic coordinating sites of the bidentate ligand can also bridge two metals via *μ*_2_-mode, forming organocopper aggregates with Cu-Cu interactions and organocuprates with contact ion pair structures. The reaction chemistry of some selected organocopper compounds is highlighted, showing their unique structure–reactivity relationships.

## 1. Introduction

Copper-mediated or -catalyzed reactions have been upgrading the toolbox of organic synthesis with a variety of cheap, efficient transformations, which benefit scientific research in a broad range of areas, such as in polymer chemistry and biochemistry [1,2,3,4,5,6,7,8,9,10,11,12]. To have a deep understanding of the reaction mechanisms, organocopper species, as proposed intermediates, are sought to be isolated. On the other hand, organocopper compounds can be easily prepared in situ and utilized as organometallic synthons to construct various small organic molecules [13,14,15]. A well-recognized example is that “hard” nucleophiles, e.g., organolithium or Grignard reagents, react with α,β-unsaturated carbonyl compounds via 1,2-addition, whereas the “soft” organocuprates will end up with 1,4-addition products with the same substrates [16,17]. In a catalytic version, in the presence of copper salts, Grignard reagents can behave similarly to organocuprates [18]. It is known that reactivities are closely connected to structural configurations. Organocopper chemistry focuses on establishing the structure–reactivity relationship of well-defined organocopper compounds, rationalizing the dissimilar reaction patterns of different organometallic copper reagents.

Transmetalation provides the most straightforward and reliable method f preparing organocopper compounds from readily available organometallic reagents [19,20], though many other synthetic strategies are also known, such as copper-halide exchange reactions [21], direct cupration [22], and the oxidative addition of reactive Rieke Cu* [23]. It should be emphasized that the identity of forming organocopper compounds is very much dependent on the ratio of starting materials. For example, in the early 19th century, Gilman et al. found that the reaction of methyl lithium and 1.0 equiv. of copper(I) salts formed insoluble methylcopper polymers as yellow solids in diethyl ether [24]. When 0.5 equiv. of copper(I) salts was used in the above condition, a clear solution of dimethyl cuprate (Gilman reagent) was created. The initial isolation and characterizations of organocopper compounds were very slow since the reactive species were subjected to thermally homolytic, oxidative, and hydrolytic decompositions [25]. A general trend is that the stability increases from alkyl, alkenyl, and aryl to alkynyl copper compounds. For example, methylcopper [24] and phenylcopper [26] clusters display rapid and slow decompositions at room temperature, respectively, while (phenylethynyl)copper [27] looks stable under ambient conditions. This stability trend could also be reflected by the numbers of single-crystal structures in the Cambridge Structural Database (CSD 5.42, 2020 November), in which roughly 185 alkyl, 288 aryl, and 326 alkynyl copper compounds have been collected. Moreover, the aggregate state, which is favorable in charge-neutral organocopper compounds, makes these polymeric compounds poorly soluble in common organic solvents. To solve the above inherent problems of organocopper compounds, coordinating heteroatom-containing moieties, such as dimethyl amino groups, were rationally introduced into the ligand skeleton by van Koten and other researches to enhance stability and solubility [28,29]. The hybrid C,N-bidentate ligands can bind to copper atoms with an enhanced chelating effect. Following a different path, Xi’s group discovered that 1,4-dilithio 1,3-butadienes (C,C-bidentate ligand) can stabilize reactive organocopper species to a great extent via a cooperative effect [30,31]. In addition, this C,C-bidentate ligand can also be regarded as an analogue of 1,2-diketimine, which turns out to be a special Z-type non-innocent ligand, producing an unprecedented aromatic dicupro-annulenes [32].

**Scheme 1:**
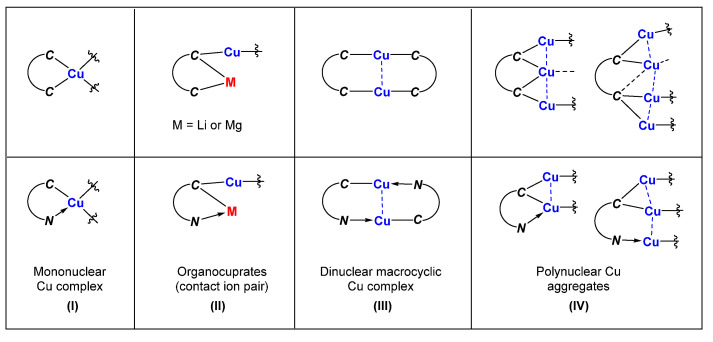
Fragments in the molecular structures with characteristic connectivity. The lines do not necessarily represent 2c-2e or 3c-2e bonds.

Here, in this review, we are interested in summarizing organocopper compounds with at least one Cu-C *σ*-bond in which the organic fragments behave as actor ligands rather than spectator ligands (e.g., Cp, NHC). For mono-dentate carbonic ligands, the binding between copper and carbon atoms normally consists of the regular two-center two-electron (2c-2e) *σ*-bonds and three-center two-electron (3c-2e) bonds [33] when the carbon atom bridges two copper atoms. In cases where the bridging carbon atom is linked with a copper and a lithium (or magnesium) atom, the binding is more likely between the 2c-2e and 3c-2e bonds. For neutral nitrogen-containing groups, the binding of Cu-N is a traditional dative bond. When extending from one coordinating site to C,N- or C,C-chelating ligands, the binding in the corresponding organocopper compounds becomes more diverse. Based on the *characteristic connectivity* of the fragments in these structures, the C,N- and C,C-chelated organocopper compounds can be put into the following four categories:

(1) Mononuclear organocopper complexes:

The two coordinating sites of the C,C- and C,N-bidentate ligand bind to the same Cu center together, forming a 5-membered chelate ring, as shown in **(I)** of Scheme 1. The two chelate rings are connected with a copper atom and will generate a spiro complex.

(2) Organocuprates with contact ion pair structures:

The two coordinating sites can be attached with different metals, with one site bound to the Cu/M atoms and the other one bound to the main group metal atoms M only. The resulting product can be regarded as being organocuprates with contact ion pair structures **(II)**.

(3) Binuclear macrocyclic copper complexes:

Each coordinating site of the C,C- and C,N-bidentate ligand can also bind an individual Cu atom with 2c-2e bonds, forming a macrocyclic dinuclear complex. As a result, each Cu atom forms a linear geometry with two units of bidentate ligands that provide only one coordinating site to each Cu atom **(III)**.

(4) Polynuclear organocopper aggregates:

If the carbanion bridges two copper atoms, the structural feature will likely be polynuclear copper aggregates with Cu-Cu interactions **(IV)**.

## 2. Mononuclear Organocopper Complexes

Although neutral N,N-chelated spiro copper complexes are relatively common, the anionic C,N- or C,C-cheated spiro counterparts are sporadic. As mentioned above, Xi’s group found that the C,C-bidentate ligand is an excellent platform to build up diversified coordination complexes across the periodic table [32,34,35,36,37,38,39,40,41,42,43,44,45,46,47,48,49,50,51,52,53,54,55,56,57,58,59,60]. The chelating effect increases the thermal stability of these organometallic species, facilitating isolation and following characterization. Additionally, their metal–carbon bonds remain reactive toward electrophiles. As a result, a good balance between stability and reactivity is achieved.

**Scheme 2:**
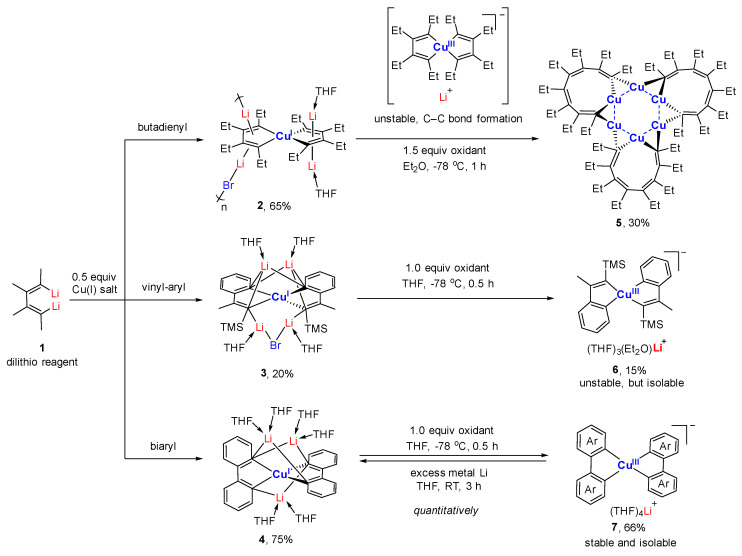
Organometallic spiro copper(I) and Cu(III) complexes [59,60].

**Figure 1:**
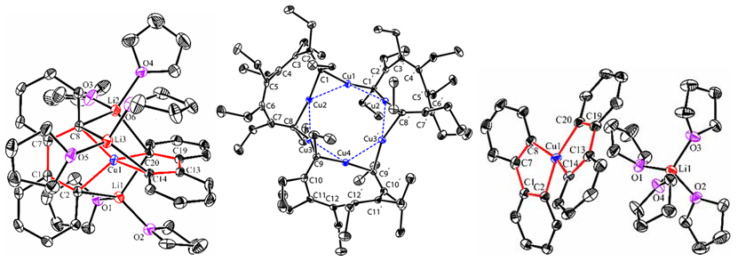
Molecular structures of **4**, **5**, and **7**. Reprinted with permission from [59,60]. Copyright 2017 and 2018 American Chemical Society.

Starting from the 1,4-dilithio 1,3-butadienes **1** (dilithio reagent for short) and 0.5 equiv. of copper(I) salts, Xi’s group isolated the first series of spiro organocopper(I) compounds **2–4** (Scheme 2) [59,60]. It seems that the terminal alkenyl carbon atom is favorable for LiBr coordination, probably because it is more electron-rich than the phenyl ones. The butadienyl spiro copper complex **2** forms one-dimensional polymers with repeating units linked by Li—Br. When one vinyl moiety is replaced by a phenyl ring, LiBr forms a Li_2_Br fragment between the two vinyl carbon atoms of **3**. No LiBr salt can be found in the biphenyl spiro copper complex **4** (Figure 1). The copper atoms are all coordinated with two dianionic ligands, forming a distorted tetrahedral geometry (dihedral angels between two five-membered chelate rings is about 63–84°). The averaged Cu(I)-C bond lengths of **2–4** are from 2.01 to 2.06 Å. Upon oxidation, either the dianionic ligand or the metal center can be oxidized depending on the electron-richness of the ligands. Compound **1** is transformed to a hexanuclear tetraenyl copper cluster **5** with a Cu_6_ hexagon. In this process, the oxidative dimerization of the original butadienyl ligand occurs. It might undergo a spiro Cu(III) intermediate, which is too unstable to be isolated. When **3** is treated with oxidizing agents, a spiro Cu(III) complex **6** is afforded and isolated successfully. Compound **6** is thermally unstable and is difficult to handle at room temperature. With an extended conjugated backbone, **4** is oxidized to form a very stable spiro Cu(III) complex **7**. In addition, **7** can be reduced by metal lithium to regenerate **4** quantitatively. Both **6** and **7** have copper atoms with a distorted square planar geometry. The averaged Cu(III)-C bonds of **6** and **7** (1.96–1.97 Å) are noticeably shorter than the Cu(I)-C bonds of **2–4**. It is noteworthy that **6** and **7** represent a novel type of unprecedented organocopper(III) complex. By virtue of Cu L_2,3_-edge X-ray absorption spectroscopy and experimentally calibrated electronic structure calculations, Lancaster and co-workers found that Cu(III) complex **7** has a LUMO (lowest unoccupied molecular orbital) with only ∼25% Cu 3d character, which suggests that the formal Cu(III) is not a physical d^8^ configuration [61].

**Scheme 3:**
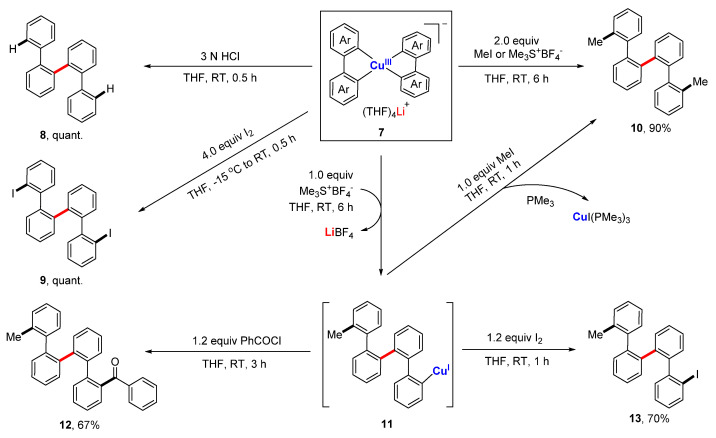
Reductive elimination of organometallic spiro Cu(III) complexes.

As revealed by the crystal structure of **7**, all four aryl rings are at *cis*-positions, which, in principle, provides a favorable arrangement for reductive elimination. However, no C-C bond formation occurred when the anionic compound **7** itself was refluxed overnight. However, when treated with electrophiles, e.g., aqueous HCl solution, iodine, and alkylation reagents, **7** afforded the symmetrical quaterphenyl derivatives **8–10** facilely in almost all quantitative yields via an electrophilic attack followed by a reduction elimination (Scheme 3). A stepwise experiment showed that **7** reacted with 1.0 equiv. of methylation agent, producing a quaterphenylcopper(I) species **11**, which can couple with other electrophiles to form the di-substituted quaterphenyl compounds **10**, **12**, and **13** in high yields. The reductive elimination of high-valent copper compounds has often been proposed as the final step to release the product, but there has been nearly no concrete experimental evidence of that until this work.

**Scheme 4:**
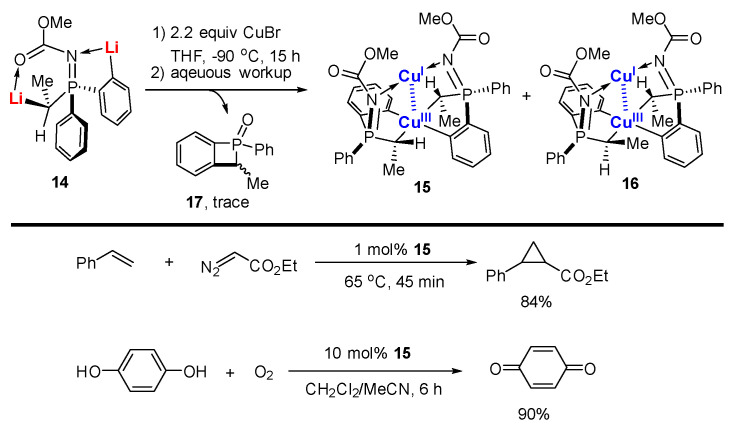
Mixed-valent bimetallic Cu(I)/Cu(III) compounds [62].

**Figure 2:**
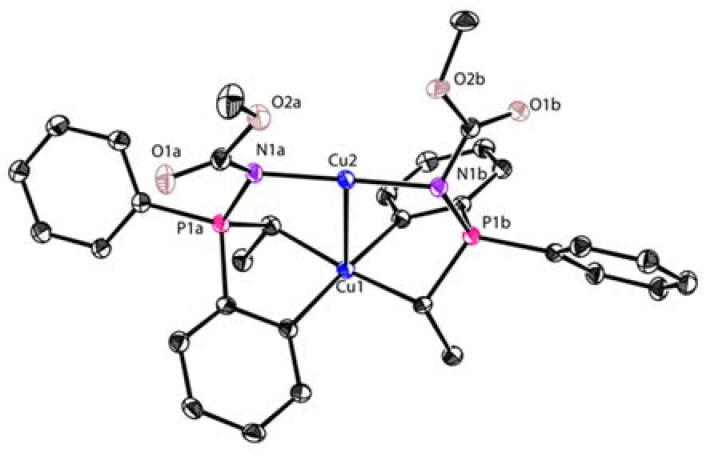
Molecular structure of **15**; drawn based on [62].

In a similar vein, López-Ortiz and co-workers reported the reaction between dilithiated phosphazene **14** with 2.2 equiv. of CuBr followed by an aqueous workup to afford two diastereotopic isomers of mixed-valent Cu(I)/Cu(III) complexes **15** and **16** in 53% combined yield, in which the Cu(III) center was chelated with two C,C-bidentate aryl-alkyl ligands (Scheme 4) [62]. A byproduct with a four-membered ring **17** was isolated as well. Both **15** and **16** can be purified by column chromatography. Accordingly, they are stable in air and moisture. Cu(III) and Cu(I) adopt a distorted square planar and linear geometry, respectively (Figure 2). The averaged Cu(III)-C bond (1.98 Å) in **15** and **16** is similar to that in **6** and **7**. The oxidation states of the Cu ions in **15** were further confirmed by extended X-ray absorption fine structure (EXAFS). Furthermore, all of the pieces of evidence, including the short Cu-Cu distance (2.74 Å) in the single-crystal structure, a critical bond point in the topological analysis of the X-ray experimental Fourier map as well as a diagnostic absorption band at 343 nm, strongly supported a weak metal–metal d^8^-d^10^ bonding between two Cu ions. Compound **15** can be used as a catalyst in catalytic cyclopropanation and aerobic oxidations. The characteristic signal of **15** has been observed and suggests that **15** remains unaffected during these transformations, probably due to its rigid structural configuration and extremely high stability.

**Scheme 5:**
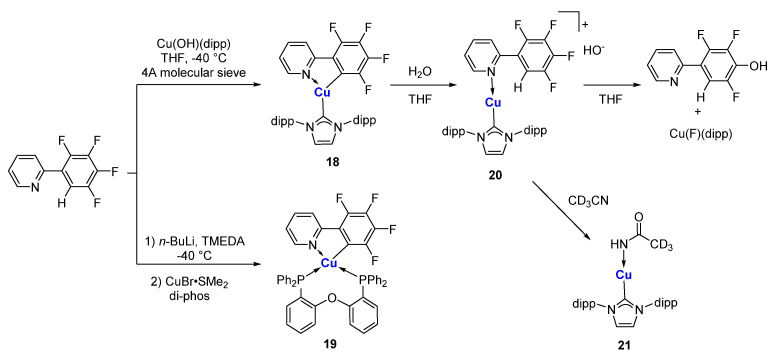
C,N-chelated organocopper compounds [63].

The compound 2-phenylpyridine and its derivatives are among the most popular scaffolds for photoactive transition metal complexes. Partial fluorination of the aryl ring makes the C-H bond at the 2-position very acidic. Additionally, at the same time, it increases the stability of the resulting Cu-C bond. Steffen and co-workers reported that the C,N-chelated organocopper compound **18** was obtained from a direct metalation from Cu(OH)(NHC), in which 4A molecular sieves play an essential role in removing water (Scheme 5) [63]. In the presence of water, compound **18** will inevitably undergo a hydrolysis process, regenerating the C-H bond and forming an ionic species **20**. The counteranion of hydroxide in **20** will replace a fluoride at the 4-position via a nucleophilic aromatic substitution in THF to 4-hydroxyl-2-arylpyridine and copper fluoride species. When MeCN is used as the solvent, hydroxide will attack MeCN, forming a copper acetamide species **21**. Similarly, a spiro complex **19** can also be prepared from lithiation and subsequent transmetalation with CuBr·SMe_2_ in the presence of diphosphine ligands. It has been shown in single crystal structures that **18** and **19** adopt distorted trigonal and tetrahedral coordination geometries, respectively. In solution, **18** is weakly emissive. In the solid state, the C,N-chelated organocopper compounds exhibit intense orange-red luminescence (λ_max_ = 610 (**18**), 607 (**19**) nm) at room temperature and have phosphorescence lifetimes of τ = 8.6 (**18**) and 9.5 (**19**) μs.

## 3. Organocuprates with Contact Ion Pair Structures

**Scheme 6:**
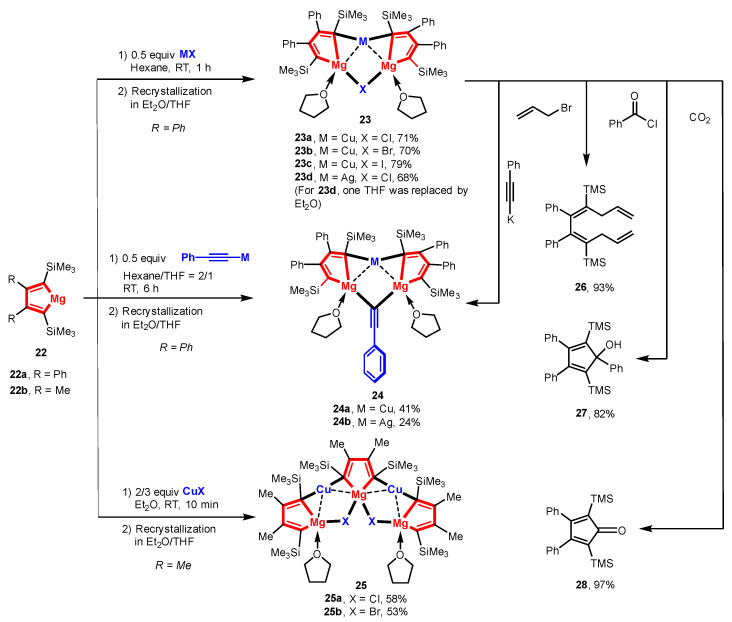
Rigid magnesium organocuprates [58].

**Figure 3:**
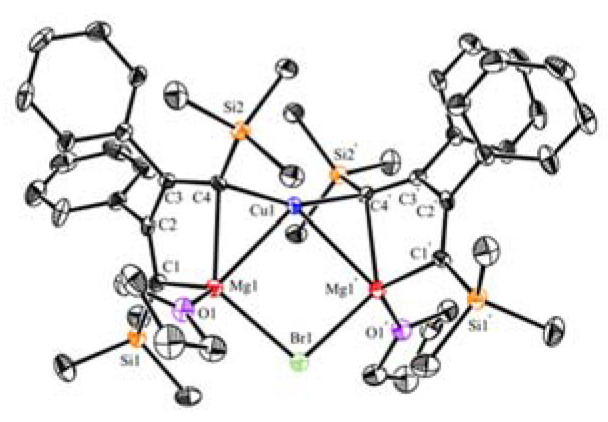
Molecular structure of **23b**. Modified with permission from [58]. Copyright 2016 John Wiley and Sons.

As mentioned in the Introduction, organocuprates have been extensively used in organic synthesis. Although numerous lithium organocuprates have been structurally characterized, the equally important magnesium organocuprates remain less unexplored, especially in terms of their well-defined structures. Only a few examples of aryl magnesium organocuprates have been previously reported [64]. As reported in the previous work of Xi and co-workers [35], magnesiacyclopentadienes **22**, the first alkaline-earth metallocyclopentadienes, have a small C-Mg-C bite angle and a coplanar 5-membered chelate ring. The bridging character of anionic carbon atoms (Lewis base) and the coordinative unsaturation of the Mg atom (Lewis acid) make **22** available to bind other metal salts. Two equiv. of **22** reacted with 1.0 equiv. of copper halides or (phenylethynyl)copper to create magnesium organocuprates **23a**–**c** and **24a** with a Cu:Mg ratio of 1:2 (Scheme 6) [58]. In addition, inert silver halides as well as alkynyl silver compounds can apply to the above transformations, producing magnesium organoargenates **23d** and **24b**. The structures of forming organocuprates are dependent on the substituents at the 2,3-positions for some reason. When the substituents are changed from Ph to Me groups, **25,** with a Cu:Mg ratio of 2:3, was obtained as the major product. Structural analysis and DFT calculation (AIM) revealed that as the Cu-C bonds form, one Mg-C bond becomes very weak (2.34 Å), while the other one remains unchanged (2.11 Å) in **23a**. It seems that two Mg atoms provide a pre-organized position to anchor the halides. The C-Cu-C bond angle (157°) as well as the Cu-C bond lengths (1.95 Å) indicates a Cu-C-Mg 3c-2e bonding character (Figure 3). As supported by the molecular structure and DFT calculations, no bonding interaction was found between Cu-Br in **23a**. On the whole, the binding of the anionic C (sp^2^) carbon atoms to the Cu atom and the bridging of the Mg atoms by the halides leads to a formal heterolytic cleavage of the M-X bond (M = Cu, Ag; X = halide, alkynyl) in a cooperative way along with the transfer of anionic X units from M centers to Mg centers. Accordingly, **23–25** can be considered as a resting-state intermediate of the transmetalation reaction between organomagnesium reagents and coinage-metal salts. The bridging ligand can be transformed quantitatively, e.g., **23c** → **23a** or **24a**. The preliminary reactivity of these rigid magnesium organocuprates was provided by the reactions of **23c** with various electrophiles, producing small organic molecules **26–28**.

**Scheme 7:**
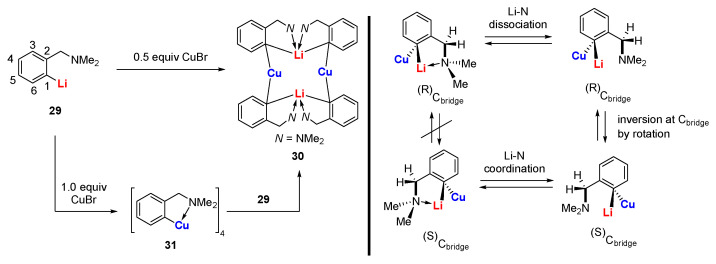
Neutral C,N-bidentate organocuprates [65,66,67,68,69].

**Figure 4:**
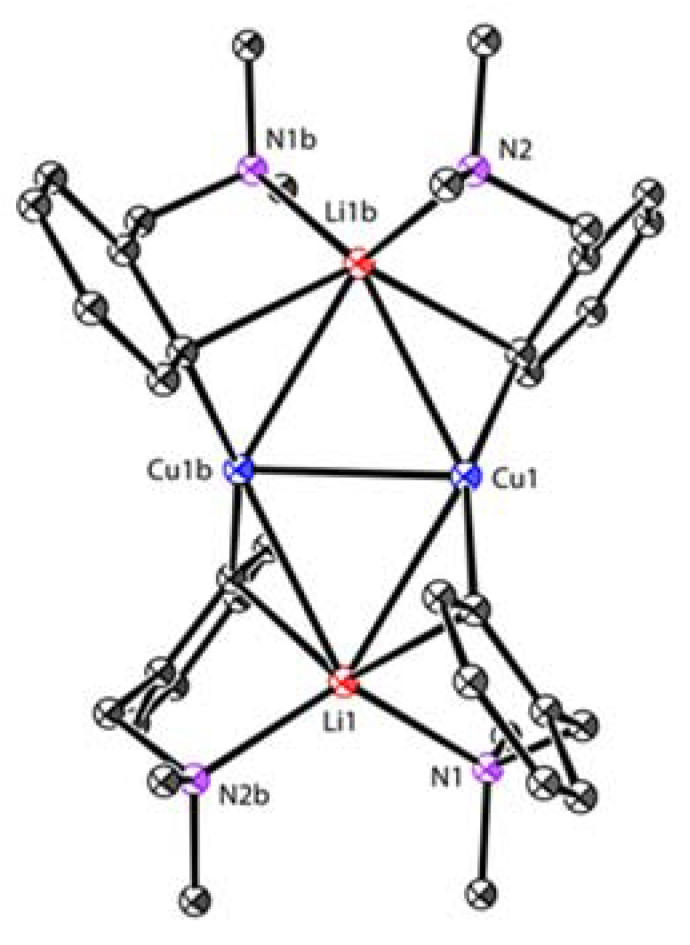
Molecular structure of **30**; drawn based on [67].

When a built-in coordinating site is added to the monoanionic phenyl skeleton at the *ortho*-position, it will generate a C,N-bidentate ligand, as shown in Scheme 7. With the (dimethylamino)methylphenyl (DMMP) lithium reagent **29** in hand, van Koten’s group developed two methods to synthesize the dicopper-dilithium organocuprate **30**: (1) a direct transmetalation of **29** with 0.5 equiv. of CuBr and (2) an interaggregate exchange reaction between DMMP copper cluster **31** (vide infra) and **29** [65,66]. The short averaged Cu-C bonds (1.94 Å) as well as the weak averaged Li-C bonds (2.39 Å) in **30** (Figure 4) suggest that the 2c-2e bond of the Cu-C bonds has a higher character [67]. However, the split coupling of ^13^C-^7^Li (7 Hz) was still observed in solution, which indicates that the s electron density is present between the Li and C (*bridge*) nuclei [68]. Meanwhile, *J* (^13^C-^7^Li) did not change during variable-temperature NMR studies, allowing it to exclude the intraaggregate exchange processes. By monitoring the characteristic signals of methylene protons (AB pattern) and N*Me*_2_ protons (two singlets) using dynamic ^1^H and ^13^C NMR spectroscopies, van Koten and co-workers found the rotation of the 3c-2e bonded aryl groups around the C(1)-C(4) axis for the first time [69]. The C_4_Cu_2_Li_2_ core fragment appears to be a persistent structural feature in neutral aryl lithium organocuprate (contact ion structure [64]).

## 4. Binuclear Macrocyclic Copper Complexes

**Scheme 8:**
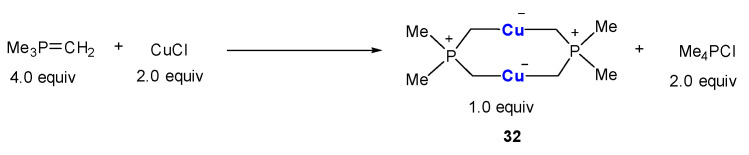
Cyclic alkyl dicopper complex [70,71].

It seems that trimethylmethylenephosphorane (Me_3_P=CH_2_) shows a better stabilization effect on the Cu-C bonds than its isoelectronic analogue Me_3_Si-CH_2_. Buchner and Zangrando found that the treatment of Me_3_P=CH_2_ with CuCl afforded a binuclear cuprate complex **32** in which negative charges are formally located at the Cu atoms while positive charges are located at the phosphine atoms (Scheme 8) [70,71]. In this process, the hybridization of CH_2_ was changed from sp^2^ to sp^3^. The core structure of P_2_(CH_2_)_4_Cu_2_ forms a coplanar 8-membered macrocycle. The averaged Cu-C bond (1.96 Å) and C-Cu-C bond angel (176°) are in the typical ranges for organocuprates.

**Scheme 9:**
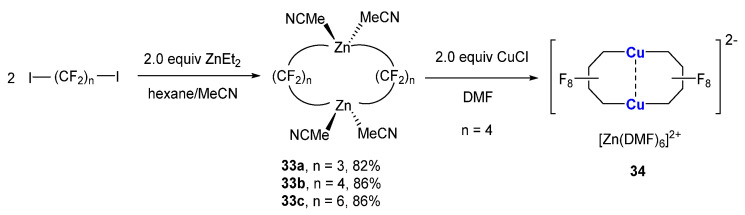
Perfluoro-dicupra-cyclodecanes [72].

Recently, there has an increasing interest in (CF_2_)_n_-containing materials in industry. However, the methods of incorporating difluoromethylene into molecules are lagging behind other perfluroalkyl counterparts. Vicic and co-workers reported a new route to access perfluoroalkyl-based metallacycles with varied ring sizes (Scheme 9) [72]. Tetra-perfluoroalkyl-dizinc compounds **33** were obtained from the reaction between diiodoperfluoroalkanes and 2.0 equiv. of ZnEt_2_. The treatment of **33b** with 2.0 equiv. of CuCl in DMF afforded a 10-membered dicopper compound **34**. The averaged Cu-C bond (1.93 Å) is relatively shorter than that of other non-fluorinated alkyl cuprates. Comound **34** was inactive towards electrophiles, which is consistent with its strong Cu-C bond and high stability.

**Scheme 10:**
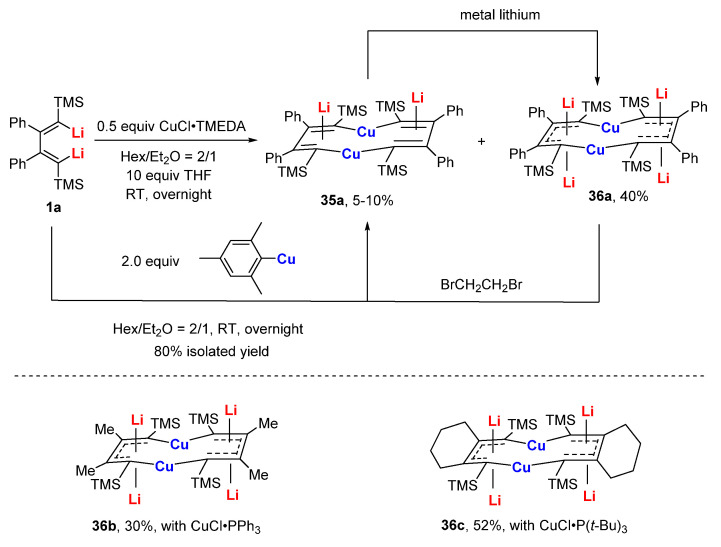
Aromatic dicupra[10]annulenes [32,47].

**Figure 5:**
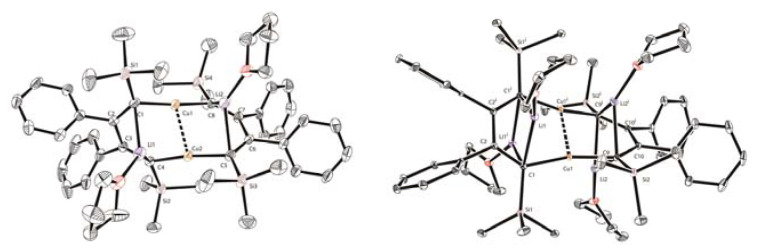
Molecular structures of **35a** and **36a**. Modified with permission from [32]. Copyright 2016 American Chemical Society.

Metal-containing aromatic molecules have attracted much attention recently. Compared to six-membered metallobezenes and metallabenzynes as well five-membered metallocylcopentadienes, macrocyclic metalla-aromatics are less explored. *Trans*,*cis*,*trans*,*cis*,*cis*-[10]annulene is a conjugate molecule with 10-*π* electrons. However, it is non-aromatic due to the steric repulsion between the two internal hydrogen atoms. It was previously envisioned that the replacement of two internal CH with a transition metal would not only release the steric hindrance but would also provide electrons to the delocalized *π*-system. Aromatic dicupra[10]annulenes **36** with four lithium atoms were first synthesized and isolated from the reaction of dilithio reagents **1** and a CuCl complex by Xi’s group (Scheme 10) [32]. In the above reactions, **35** was isolated with two lithium atoms as a small amount of byproduct that is also able to be efficiently synthesized from dilithio reagent and mesitylcopper. More importantly, reversible two-electron transformations between **35** and **36** were observed [47]. Compared to the alternating bonds of the butadienyl skeleton in **35a** (1.52, 1.37 Å), the C-C bonds of annulene moiety in **36a** are remarkably averaged (1.47, 1.42 Å), suggesting a considerable π-delocalization (Figure 5). The Cu-C bonds of **36a** (1.92 Å) were notably shorter than those of **35a** (1.96 Å), which might be attributed to the extra two electrons posed to **36a**. The Cu 2p_3/2_ binding energy (932.9 eV) and Cu LMM kinetic energy (915.8 eV) of **36b**, as displayed by XPS data, show that the Cu atoms in **36** are more likely to be Cu(I). With the assembled experimental results (the above crystallographic data; low-frequency resonance signals of ^7^Li NMR: −5.1 to −6.2 ppm) and DFT calculations (negative values of ISE: −11.7 and −21.5; large negative NICS values: −9.0 to −12.8), it is safe to conclude that dicupra[10]annulenes are aromatic. AdNDP analysis has previously suggested dicupra[10]annulenes could be regarded as a 10-π aromatic system. Based on independent theoretical calculations, Grande-Aztatzi and co-workers consider dicupra[10]annulenes to be a metalla-naphthalene [73]. Zhu and co-workers suggest that dicupra[10]annulenes have 16e Craig-type Möbius aromaticity [74]. Additionally, both Zhu’s and Sundholm’s calculations reveal that lithium counterions play an important role in these organocopper complexes [74,75].

**Scheme 11:**
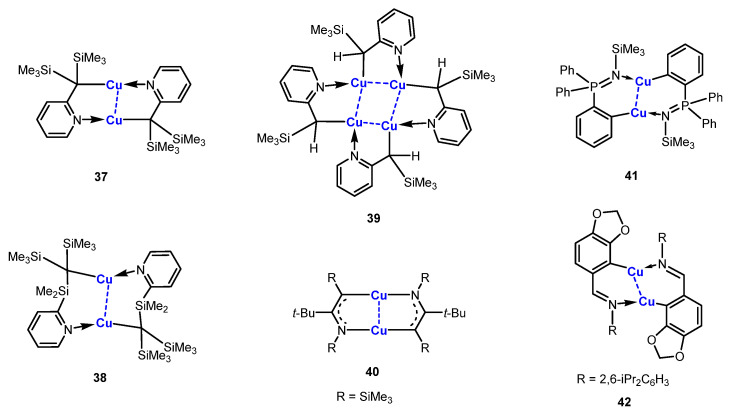
C,N-chelated cyclic organocopper compounds [76,77,78,79,80,81,82].

As shown in Scheme 11, the C,N-chelated dicopper compounds **37**,**38** and **40–42** documented in the literature are generally macrocyclic neutral species since the two negative ligand charges are exactly balanced with two Cu(I) atoms [76,77,78,79,80,81,82]. An early example of structurally well-defined alkylcopper compounds was [Me_3_SiCH_2_Cu]_4_, in which trimethylsilyl groups not only pose steric hindrance to increase the stability but also improve the solubility by virtue of the lipophilicity of the silicon groups [83,84]. White and others found that incorporating an *ortho*-bulky bis-trimethylsilyl-alkyl ligand at a pyridine can stabilize t dicopper compound **37** to a great extent [76,80]. Compound **37** can be sublimated at 160 °C without decomposition. Compund **37** features an 8-membered core structure (C_4_N_2_Cu_2_). When increasing the distance between the alkyl moiety and the pyridine, a dicopper compound **38** with a 10-membered ring can also be generated, as reported by Eaborn and Smith [81]. However, with a smaller steric effect, the alkyl-pyridine ligands will bind four copper atoms, generating a tetracopper complex **39** [77]. Similarly, three dicopper compounds **40–42** based on 1-azaallyl, aryl-phosphanimine, and aryl-imine ligands were synthesized from the corresponding lithiated starting materials and copper salts by Lappert [78], Stalke [79], and Schmidt [82], respectively. The Cu-C bonds of **37–40** are in the range of 1.93–1.95 Å, which suggests a mostly 2c-2e Cu-C bond, while the Cu-C bonds (1.91 Å) in **41** and **42** fall in the short end of the range observed for Cu-C 2c-2e bonds. Cyclic voltammetry and bulk electrolysis of **37** show very promising evidence of the existence of formal copper(II) alkyl species with relatively high stability.

## 5. Polynuclear Organocopper Clusters

**Scheme 12:**
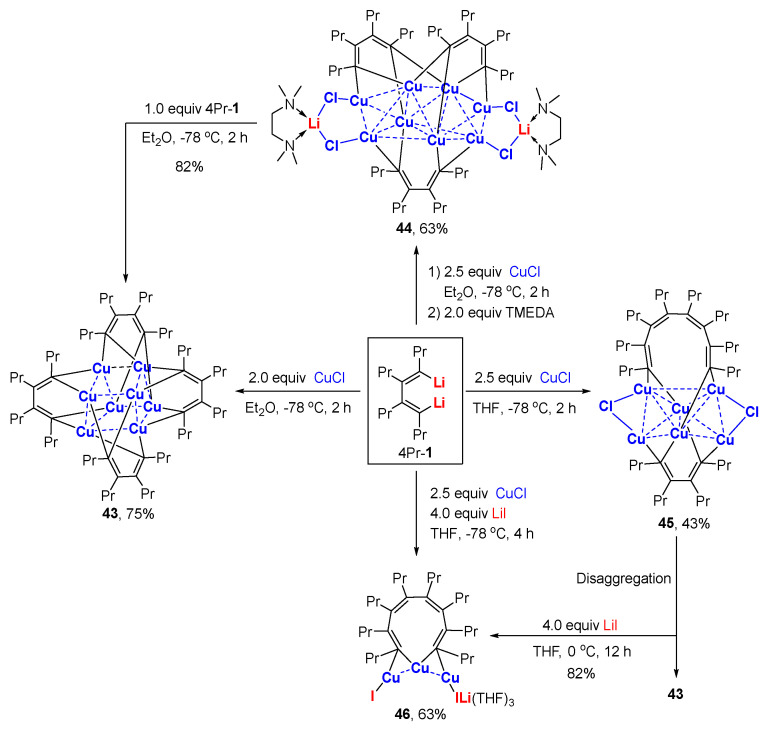
Conjugate C,C-bidentate organocopper aggregates [56].

**Figure 6:**
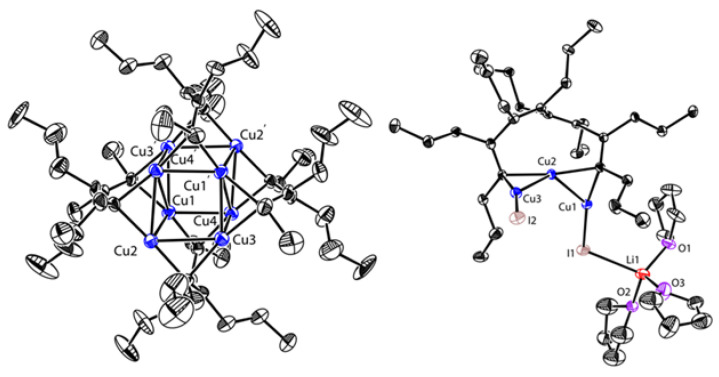
Molecular structures of **35a** and **36a**; drawn based on [56].

Fifteen years ago, Xi and co-workers reported that the *cis*-1,3-butadienyl 1,4-dicopper species, obtained from the reaction beteen dilithio reagent and 2.0 equiv. of CuCl, efficiently produced multi-substituted semibullvalenes after thermolysis at 50 ℃ in non-coordinating toluene solvent [85]. Later on, it was found that the same copper species underwent lithium iodide-assisted linear dimerization in Et_2_O at 0 °C to afford an all-*cis* octatetraenyl dicopper intermediate that could be subject to subsequent Pd-catalyzed cross-coupling reactions with halides [86]. In order to unveil the nature of butadienyl copper species, Xi and co-workers carefully conducted transmetalation reactions at a low temperature (−78 ℃) in order tto avoid uncontrollable thermal decompositions. The reaction of the dilithio reagents 4Pr-**1** with 2.0 equiv. of CuCl in Et_2_O afforded 1,4-dicopper tetramer **43** at a 75% yield (Scheme 12) [56]. When 2.5 equiv. of CuCl was used in the presence of 2.0 equiv. of TMEDA, a different copper aggregate **44** was obtained at a 63% yield. Compound **44** could be transformed to **43** through treatment with 1.0 equiv. of dilithio reagent. Additionally, the products were also dependent on the solvents and additives, e.g., lithium salts. When THF was used as a solvent, the reaction between dilithio reagent and 2.5 equiv. of CuCl produced **45**, which was linked by a 1,3-butadienyl and 1,3,5,7-octatetraenyl units at a 43% yield. In the presence of extra LiI, the above reaction produced the 1,3,5,7-octatetraenyl-1,8-dicopper compound **46** at a 63% yield. The unit of 1,3,5,7-octatetraenyl was formed from the dimerization of butadienyl copper complexes, as observed in previous work [86]. Compounds **46** and **43** can also be generated from the reaction between **45** and LiI. Structural analysis of **43–46** reveals that the Cu-C bonds and C-Cu-C bond angles are in the range of 1.96–2.08 Å and 73–84°, respectively. The close Cu-Cu distances (2.37–3.01 Å) suggests weak d^10^-d^10^ interactions in all of the copper aggregates above (Figure 6).

**Scheme 13:**
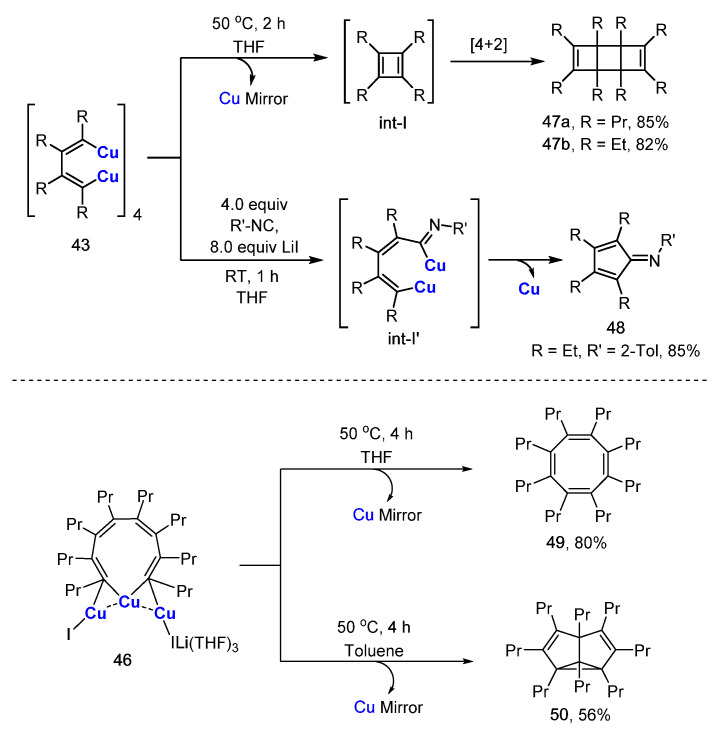
Reactivity of bis-enyl organocopper aggregates.

The reactivity of the above bis-enyl copper compounds was displayed by the reactions of **43** and **46** (Scheme 13). Compound **43** produced tricycle-octa-diene **47** and cyclopenta-2,4-dien-1-imine **48** via thermolysis and the insertion of isocyanide, respectively. When heated in THF, **46** afforded multi-substituted cyclooctatetraene **49**. In toluene, the thermolysis of **46** produced semibullvalene **50** as the major product, which was consistent with earlier work [85].

**Scheme 14:**
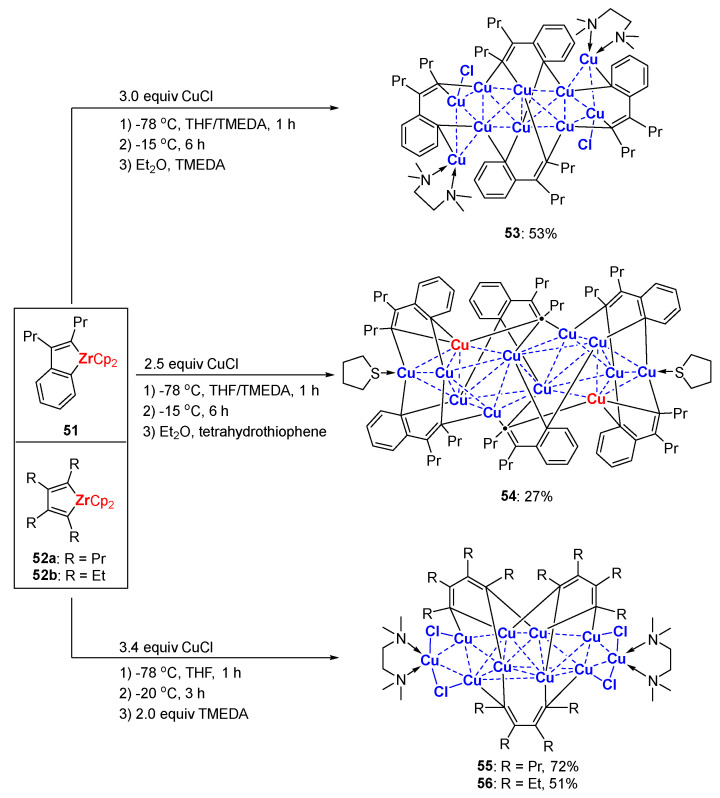
Styrenyl and budadienyl organocopper aggregates [57].

**Figure 7:**
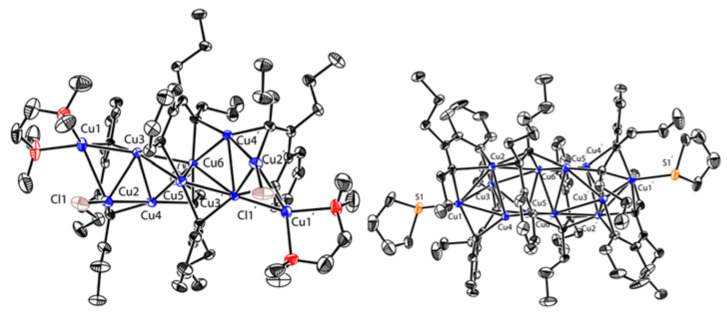
Molecular structures of **53** and **54**; drawn based on [57].

Copper-mediated reactions of zirconacyclopentadienes (or zirconaindenes) and electrophiles developed by Takahashi and others are extensively used to construct a variety of functional organic molecules [87,88]. However, organocopper intermediates remain elusive though the methodologies were reported about twenty years ago. Inspired by this work, Xi’s group revisited the transmetalation reaction between zirconium reagents and copper salts. Salt-free zirconaindenes **51** and zirconacyclopentadienes **52** were readily prepared according to the reported procedure [87]. A 1:3 reaction of **51** and CuCl in THF/ TMEDA afforded a tetrameric styrenyl copper aggregate **53** at a 53% isolated yield (Scheme 14) [57]. When **51** was treated with 2.5 equiv. of CuCl, a hexameric styrenyl copper aggregate **54** was obtained at a 27% isolated yield after crystallization in Et_2_O and tetrahydrothiophene (THT). Zirconacyclopentadienes **52a**,**b** reacted with 3.4 equiv. of CuCl in THF, affording trimeric butadienyl copper aggregates **55** and **56** at 72% and 51% yields, respectively. The additional ancillary ligand (TMEDA vs. THT) could change the aggregate state of the styrenyl clusters. In terms of molecular structures, **53** consists of ten copper atoms, while **54** is a hexanuclear cluster with twelve copper atoms. The lengths of the Cu-C(alkenyl) bonds (1.98–2.01 Å) are comparable to those of the Cu-C(aryl) bonds (1.98–2.02 Å), as shown in **53** (Figure 7). The binding mode of **54** looks more complicated and also more unique. Compound **54** comprises two unusual *μ*_3_-alkenyl carbon atoms (black dots: C17, C17′) and the corresponding two tri-coordinated copper atoms (red: Cu2, Cu2′). Compounds **55** and **56** share identical core copper structures. In addition, **55** and **56** can be regarded as variants of **44** by replacing the two lateral lithium atoms with copper atoms. The substituents of the butadienyl skeleton seem to have an influence on the stability of their copper clusters. In Et_2_O, **55** is relatively stable below 10 ℃, whereas **56** is would thermally decompose and release the copper mirror at 10 ℃ within 0.5 h. The reactivity of **55** was provided by its coupling reaction with diiodobenzene, producing multi-substituted naphthalene.

**Scheme 15:**
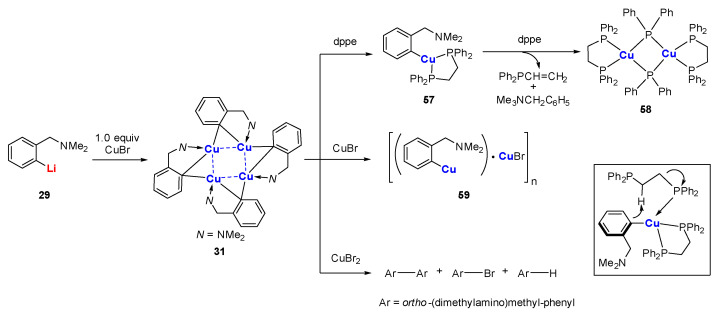
Synthesis and reactivity of C,N-chelated tetranuclear organocopper cluster [89,90,91,92,93,94,95].

**Figure 8:**
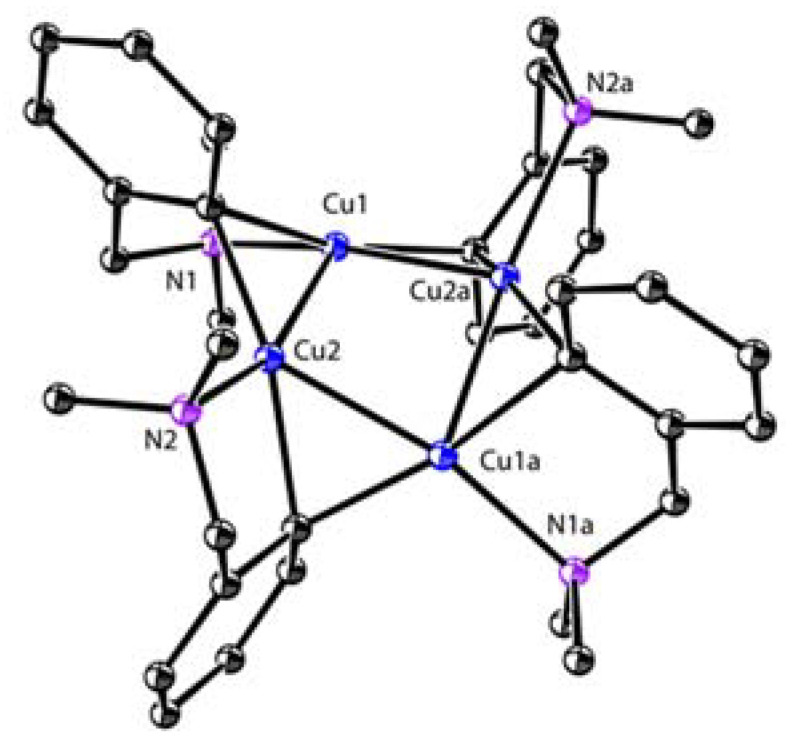
Molecular structure of **31**; drawn based on [92].

The intramolecular stabilization effect developed by van Koten was initially used to synthesize organocopper clusters. The reaction of **29** with 1.0 equiv. of CuBr afforded a LiBr-free tetranuclear copper cluster **31**, which is thermally stable up to 170–185 ℃ (phenylcopper: slow decomposition at room temperature) [89,90]. The molecular structure of **31** comprises four copper atoms in a butterfly arrangement (Scheme 15) [91,92]. Each C_ipso_ bridges two copper atoms via the 3c-2e Cu-C bonds (1.97–2.16 Å). Each copper atom has a distorted trigonal planar geometry (Figure 8). Variable temperature ^1^H NMR spectroscopy shows that the methylene and N*Me*_2_ signals are singlets that are independent of the temperature, which suggest a weak Me_2_N→Cu interaction in solution. Whereas the reaction between **31** and 1.0 equiv. of dppe afforded a 1:1 donor–acceptor copper complex **57**, the addition of excess dppe resulted in an unusual C-P bond cleavage with the formation of a phosphidocopper complex **58**, Ph_2_PCH=CH_2_, and Ar-H [93,94]. This process can be explained by a concerted mechanism, as shown in the box. A 1:1 reaction of **31** with CuBr affords a polymeric complex **59**. When cupric halides were used instead, the oxidative coupling products Ar-Ar, Ar-X, and Ar-H were obtained [95]. In addition, van Koten and co-workers reported a serial of organocopper aggregates with the N-C-C-C-Cu connectivity supported on other C,N-bidentate ligands, such as 8-(dimethylamino)naphthyl [96], 2-oxazoline-4-methylphenyl [97,98], and (2-(dimethylamino)phenyl)-1-vinyl [99,100,101,102].

**Scheme 16:**
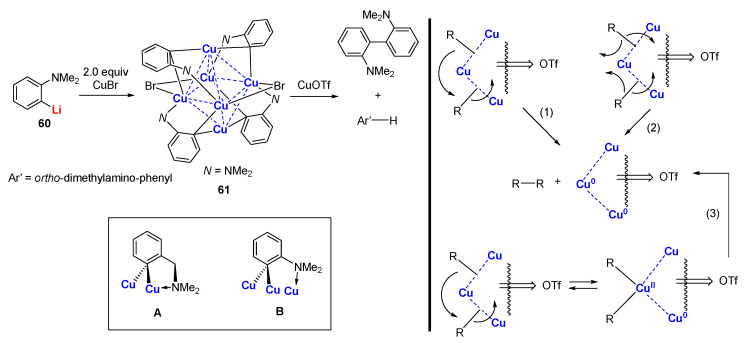
C,N-chelated hexanuclear organocopper cluster and reactivities [103,104,105,106,107,108].

When a built-in heteroatom moiety is directly attached to the phenyl skeleton, the C,N-bidentate ligand displays different features when binding to the copper atoms (N-C-C-Cu connectivity). Van Koten reported that the treatment of **60** with copper bromide affords a hexanuclear copper cluster **61** that includes six copper atoms in a distorted octahedral arrangement (Scheme 16) [103,104,105,106]. Each bromide atom bridges the *trans*-equatorial edges. Each C,N-bidentate ligand bridges three copper atoms on the face of an octahedron. Molecular weight determinations and ^1^H NMR spectroscopy supported that **61** retained its aggregate structure in solution. Me_2_N was observed as two broadened singlets (1.84, 2.92 ppm) at room temperature, suggesting a relatively strong Me_2_N→Cu bonding interaction in contrast to the one seen in **31**. When the temperature increases to 90 ℃, the two singlets coalesced to a single singlet (2.42 ppm). In **31**, the C_ipso_ atom and Me_2_N bind to the same copper atom, leading to a 5-membered chelate ring (**A**), whereas in **61**, Me_2_N→Cu coordination takes place with a different copper atom to which the aryl group is not bonded (**B**), which is due to the steric effects. When **61** was treated with CuOTf, a selective, quantitative coupling of biaryls Ar’-Ar’ occurred, which was unexpected [107,108]. Only a trace amount of Ar’-H was formed, which excluded the pathway involving free radicals. The charge transfer from the core coppers to the strongly electron accepting OTf groups, which reduces the electron density of the core skeleton, accounts for the C-C bond formation. The proposed mechanism (3), including the valence disproportionation inside the metal core followed by the reductive elimination of Ar’-Ar’, rationalized the large influence of the identity of the anions.

**Scheme 17:**
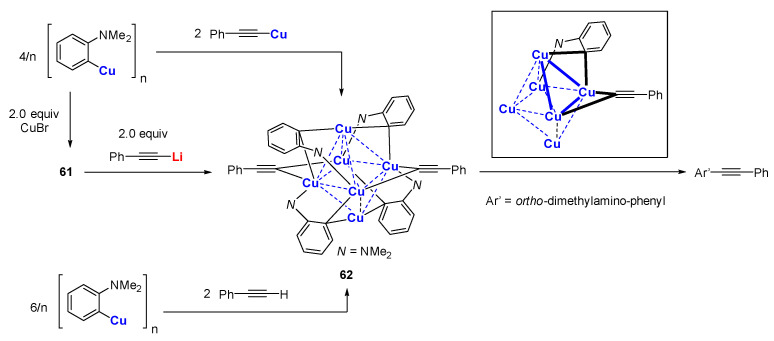
Selective coupling from the thermolysis of hexanuclear organocopper cluster [109,110,111,112].

**Figure 9:**
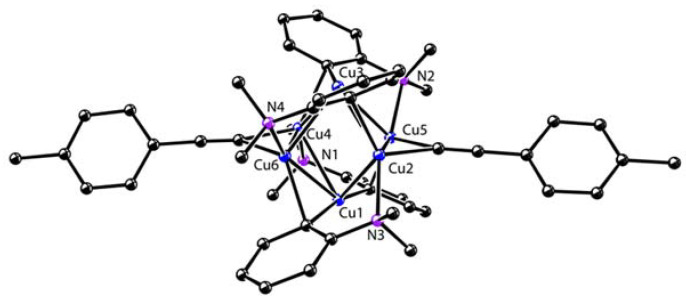
Molecule structure of **62**; drawn based on [109].

Based on the earlier work shown in Scheme 16, van Koten reported that **62** could be synthesized via three methods (Scheme 17) [109,110,111,112]. The thermolysis of Ar’_4_(CCPh)_2_Cu_6_ **62** results in the exclusive formation of the mixed coupling product Ar’-CCPh. The absence of Ar’-H or PhCC-H from hydrogen abstraction indicates that the reaction undergoes a concerted pathway rather than proceed via free radicals. The molecular structure of **62** comprises exclusively triangular copper faces occupied by one Ar’ and one PhCC ligand, whereas Cu_3_Ar’_2_ or Cu_3_(CCPh)_2_ faces are absent, explaining the high selectivity of the asymmetric C-C bond formation (Figure 9).

## 6. Conclusions and Perspective

In this review, we have summarized the structurally well-defined organocopper compounds based on C,N- and C,C-bidentate ligands, from mononuclear copper spiro compounds to polynuclear copper clusters. It was shown that the intramolecular stabilization effect of the C,N-bidentate ligands and the cooperative effect of the C,C-bidentate ligands (*cis*-1,3-butadienyl) can both enhance the solubility and stability of the resulting organocopper compounds. In the molecular structures, copper(I) atoms adopt linear, trigonal, or tetrahedral geometries, depending on the coordination numbers. In charge-neutral copper aggregates, the electron-deficient 3c-2e Cu-C bonds are predominant, whereas in more electron-rich organocuprates, the Cu-C bonds have a much higher degree of 2c-2e bond character.

The strategy for enhancing the intramolecular coordination was further developed by adding two build-in heteroatom moieties to the aryl ring. Consequently, the original ligands turn into tridentate pincer ligands, e.g., C,N,N and N,C,N-ligands [29], which are now widely used in organic synthesis and small molecules activations. Another point of interest in organocopper chemistry is high-valent organocopper compounds [113]. It was found that the macrocyclic ligands with three intramolecular heteroatom moieties, e.g., C,N,N,N-cyclic ligands, were excellent platforms to support organocopper(III) compounds. Moreover, there has been increasing interest in more challenging organocopper(II) compounds in recent years since many Cu-catalyzed reactions may have Cu(II)-C containing species as their intermediates.

## Data Availability

Not applicable.
